# 3D Analysis of Deformation and Porosity of Dry Natural Snow during Compaction

**DOI:** 10.3390/ma12060850

**Published:** 2019-03-13

**Authors:** Lavan Kumar Eppanapelli, Fredrik Forsberg, Johan Casselgren, Henrik Lycksam

**Affiliations:** Division of Fluid and Experimental Mechanics, Luleå University of Technology, 971 87 Luleå, Sweden; fredrik.forsberg@ltu.se (F.F.); johan.casselgren@ltu.se (J.C.); henrik.lycksam@ltu.se (H.L.)

**Keywords:** tomography, micro-CT, snow grains, digital volume correlation, snow microstructure, snow properties

## Abstract

The present study focuses on three-dimensional (3D) microstructure analysis of dry natural snow during compaction. An X-ray computed microtomography (micro-CT) system was used to record a total of 1601 projections of a snow volume. Experiments were performed in-situ at four load states as 0 MPa, 0.3 MPa, 0.6 MPa and 0.8 MPa, to investigate the effect of compaction on structural features of snow grains. The micro-CT system produces high resolution images (4.3 μm voxel) in 6 h of scanning time. The micro-CT images of the investigated snow volume illustrate that grain shapes are mostly dominated by needles, capped columns and dendrites. It was found that a significant number of grains appeared to have a deep hollow core irrespective of the grain shape. Digital volume correlation (DVC) was applied to investigate displacement and strain fields in the snow volume due to the compaction. Results from the DVC analysis show that grains close to the moving punch experience most of the displacement. The reconstructed snow volume is segmented into several cylinders via horizontal cross-sectioning, to evaluate the vertical heterogeneity of porosity distribution of the snow volume. It was observed that the porosity (for the whole volume) in principle decreases as the level of compaction increases. A distinct vertical heterogeneity is observed in porosity distribution in response to compaction. The observations from this initial study may be useful to understand the snow microstructure under applied stress.

## 1. Introduction

The three-dimensional (3D) arrangement of ice crystals and pores, i.e., the microstructure of snow, changes with time due to exchanges of matter between the ice crystals. Although the link between the snow microstructure and its physical properties has been addressed for a long time [1,2], it is still difficult to characterize the snow microstructure and its evolution over time. Two common approaches used to characterize the 3D microstructure of snow are serial sectioning [3,4] and X-ray computed microtomography (micro-CT) imaging [5,6]. The micro-CT is a technique for non-destructive 3D imaging of internal microstructures [7]. Based on the acquired 3D data it is possible to make a quantitative analysis of internal features such as porosity, cracks, grains, fibres etc., as well as material deformation and strain [8]. In addition, the micro-CT enables the evolution of a material microstructure to be studied in both temporal and spatial domain.

The micro-CT method has been used by many researchers for more than ten years to visualize the snow microstructure. However, the 3D quantitative analysis of displacement of snow grains during compaction is limited. Schleef and Löwe [9] addressed the influence of external mechanical stress on isothermal densification and specific surface area (SSA) of snow, using the micro-CT measurements. They reported that evolution of the snow SSA is independent of the snow density, while snow densification increases with increasing external stress. Kaempfer and Schneebeli [10] investigated the isothermal metamorphism of fresh snow at different temperatures for nearly one year. They deduced snow microstructural parameters from the tomographic images, which describe the structural information related to grain boundaries. Pinzer et al. [11] performed time-lapse micro-CT experiments on snow metamorphism under a static temperature gradient. They observed the structural evolution and mass transfer within snow through ice grains. Ebner et al. [12] further observed the snow metamorphism exposed to an advective airflow and reported that saturated airflow has no influence over grains coarsening rate. Wang and Baker [13] investigated the snow microstructure evolution under compression tests, based on the X-ray micro-CT imaging. They also performed analysis of SSA, structure model index and structure thickness. One of their findings from interrupted compression tests was that the SSA of snow decreased more rapidly than the determined values of SSA. Kerbrat et al. [14] and Hagenmuller et al. [15] proposed image processing techniques to determine snow properties such as density and SSA based on the micro-CT measurements. They emphasized that the retrieval method of these snow properties is sensitive to the voxel size (10 μm for their experiments), especially for fresh snow. Wiese and Schneebeli [8] performed the micro-CT measurements of snow microstructure under the influence of settlement at constant temperature gradient. Their observations show an increase in density, strain and viscosity over time due to settlement induced via external loading. All these investigations have larger focus on microstructural parameters, and smaller focus on structural changes in a snowpack at granular level due to compaction. Further, Schleef et al. [16] observed the impact of various levels of compression on the microstructure of snow, which is similar to the presented study. The major focus of their study was on the dependence of density and SSA to the applied stress using a microcompression device. However the presented study focuses on the characteristics of individual ice crystals under external loading. A high quality tomographic data with spatial resolution of 4.3 μm was carried out in this study to observe the microstructural features of snow grains with respect to applied stress.

The purpose of this initial study is to analyze the 3D images of snow grains during compression tests induced via in-situ uniaxial load. The presented analysis of the micro-CT measurements focuses on displacement and strain fields based on digital volume correlation (DVC) [17] and porosity distribution of the investigated snow volume. Microstructural parameters such as density and SSA have been calculated for various load stages from the tomography data. The investigated snow volume is prepared from a freshly fallen low density snow that was collected right after precipitation. Section 2 focuses on experimental arrangement and measurement procedure. Section 3 describes the applied techniques, which are DVC and porosity analysis. Section 4 details the observations of the study and discussions of the observed results are given in Section 5. A summary including conclusions are presented in Section 6.

## 2. Experimental Procedure

### 2.1. Micro-CT System with In-Situ Load Module

**Figure 1:**
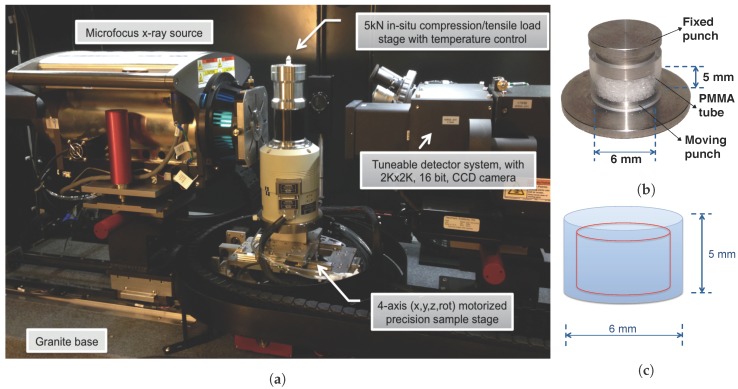
Experimental arrangement of the micro-CT system: (**a**) the micro-CT consists of a sealed microfocus X-ray tube, 4-axis sample stage, a photo detector and a temperature controlled in-situ load stage; (**b**) the sample holder was 6 mm in diameter and 5 mm in height, note that the material visible in the sample holder is sugar; (**c**) selection of volume of interest (VOI) in the snow volume.

Snow sampling and scanning were performed at the micro-CT lab, Luleå University of Technology, Sweden. The 3D images of snow microstructure were obtained using a ZEISS Xradia 510 Versa (Carl Zeiss X-ray Microscopy, Pleasanton, CA, USA). The Xradia 510 Versa can achieve 0.7 μm of true spatial resolution with minimum achievable voxel size of 0.07 μm. The components of the micro-CT system are a sealed microfocus X-ray tube, 4-axis sample stage, a photo detector and a load stage, see Figure 1a. The system is equipped with a Deben CT5000TEC temperature (Deben UK Limited, Bury Saint Edmunds, UK) controlled load stage with a 500 N load cell. The Xradia 510 Versa can be operated at tube voltage range of 30–160 kV with maximum output of 10 W.

A sample holder (Figure 1b) was specifically designed to visualize material properties for the micro-CT experiments. This holder has a fixed punch made of brass, a Polymethyl methacrylate (PMMA) tube with inner and outer diameter of 6 mm and 10 mm, respectively. Further, a moving punch with diameter of 5.95 mm was made of aluminium. The diameter of the moving punch is slightly smaller than the inner diameter of the PMMA tube, in order to ensure a smooth and frictionless compression. The in-situ load stage was used to apply uniaxial stress to compact a snow volume via the moving punch, which means the compression is applied from the bottom, and both the punch plates have smooth and flat surface. The investigated snow sample was small enough such that it was not required to move the sample vertically during data acquisition.

Scout-and-Scan^TM^ Control Software (Carl Zeiss X-ray Microscopy, Pleasanton, CA, USA) was used for reconstructing the scanned images. Quantitative analysis of microstructure (shape of crystals, porosity etc.) was obtained from 3D image analysis, using the software Dragonfly Pro (Carl Zeiss X-ray Microscopy, Pleasanton, CA, USA) (ORS).

### 2.2. Snow Sampling

An undisturbed natural dry snow block was collected right after latest precipitation outside of Luleå University of Technology (LTU), Sweden and ambient temperature was about −4 °C at the time of collection. The acquired snow block was immediately placed in a freezer held at −18 °C. Tools such as sample holder, spatula etc., that were required to prepare a final snow sample were also kept in the freezer. After the sample holder and tools were cooled down, a cylindrical snow sample was prepared using thermal insulated gloves, still being inside the freezer. Thereafter, the sample holder with snow sample was transferred quickly from the freezer to the in-situ load stage that was held at −15 °C. Surface of the snow sample was flattened using the moving punch to minimize the skewness of the applied load.

### 2.3. Data Acquisition

The X-ray source voltage and current were set to 50 kV and 80 μA, respectively. Temperature of the in-situ load stage was maintained at −15 °C for all the measurements. To avoid the microstructural damage close to the edges, an interior VOI was defined for the DVC analysis, which corresponds to 4.4 mm in diameter and 3.9 mm in height, see Figure 1c. A total of 1601 projections of the investigated snow volume were recorded as the sample rotated over 360° in high resolution setting for a period of 6 h.

Prior to the scan, the snow sample was placed in the micro-CT chamber for 30 min to ensure thermal equilibrium for the sample and mount. Initially, a low resolution scan was carried out for 30 min to improve stability of the source and to check if the reconstruction is as expected. Thereafter, higher resolution scans of the snow volume were performed using the 4× objective and 4.4 mm field of view (FOV). In this case, the micro-CT reconstructs the spatial distribution of ice crystals and pore space with a resolution in terms of voxel size 4.3 μm at an exposure time of 12 s per projection.

**Figure 2:**
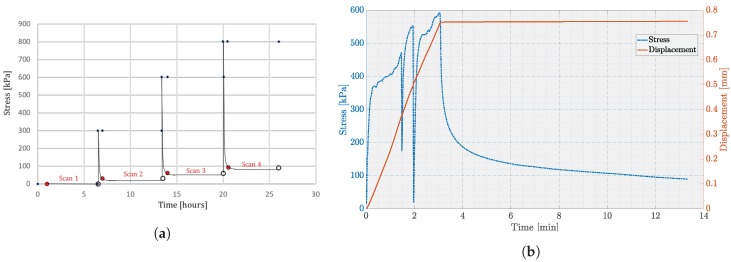
Graphical representation of compression tests: (**a**) schematic representation of the applied loading profile; (**b**) real-time force and displacement data for 0.6 MPa loading profile. In Figure 2a, red circles represent the beginning of scanning, white circles represent the end of scanning and blue circles represent the relaxation time during respective load cycles.

Figure 2a shows a schematic representation of the loading profile, where micro-mechanical uniaxial compression tests on the snow sample were performed at four load states such as 0 MPa, 0.3 MPa, 0.6 MPa and 0.8 MPa. The load stage was used in a continuous mode where the compression was applied until the user-defined load was reached. The snow volume was scanned first at 0 MPa (unloaded state) and then subsequent scans were conducted at three load states. After each loading state, the snow sample was allowed to rest for 30 min prior to the scan.

Figure 2b shows the loading and displacement data for the load state of 0.6 MPa. Figure 2b shows rapid and large deformation of snow during the loading period due to grain rearrangements, however the load profile curve was steeper up to 0.35 MPa as the snow sample was already compacted up to 0.3 MPa during the previous load state. After 0.35 MPa, snow tends to resist the force up to 0.45 MPa where the snow structure breaks due to failure of cohesive bonds and recrystallization. The similar behavior can be observed when the force starts to increase up to 0.55 MPa. After reaching the desired load, the snow sample is set to relax for 30 min prior to the scan. During the load relaxation, the stress relaxed to a residual value of about 64 kPa after 30 min. Apparently the described global structural failures (see Figure 2b) during the load profile allow the ice crystals to move into more compact arrangements, which tend to strengthen the bonds between grains [18,19]. Furthermore, there was no scan performed during the relaxation phase, as this would create motion artefacts due to high resolution.

## 3. Methods

### 3.1. DVC

The DVC technique is used to quantify the internal displacements and strain field throughout a sample volume. This technique focuses on movement and re-distribution of microstructural features of the sample volume in response to compaction via e.g., uniaxial stress. A complete description of the DVC technique can be found in [20,21,22].

The DVC technique requires volume images of a sample in reference (unloaded) and deformed (loaded) states. These volume images are then divided into sub-volumes that are independently correlated, and mapped into global deformation and strain fields. The DVC analysis was carried out using the software LaVision Davis (LaVision Inc., Ypsilanti, MI, USA) 8.4, where a multi-grid differential correlation approach was used, with final sub-volume size of 32 × 32 × 32 voxels and 75% overlap.

### 3.2. Porosity, Density and SSA

The reconstructed 3D images were first filtered by a median filter to remove noise in the images. After filtering, the volume data was segmented using a threshold obtained with the Otsu method [23]. The filtering and thresholding algorithms segment the grayscale images to binary images containing the voxels composed of either ice or air. Furthermore, these binary images were then used to determine density, SSA and porosity distribution of the snow volume. The Otsu segmentation method was previously shown to be subject to systematic bias in the estimation of density and SSA [15].

Porosity can be defined as the ratio between the volume of the pore space (V${}_{e}$) to the total volume of the sample (V${}_{t}$). In order to determine the volume of pore space, first volume of ice crystals (V${}_{i}$) is determined based on the segmented grayscale images [24,25]. Subtraction of V${}_{i}$ dataset from V${}_{t}$ dataset then gives the required V${}_{e}$ dataset and calculation of porosity is in the form

(1) $$\varphi =\frac{V_{e}}{V_{t}}*100=\frac{V_{t}-V_{i}}{V_{t}}*100\;\;\left[\mathrm{in}\;\%\right].$$

**Figure 3:**
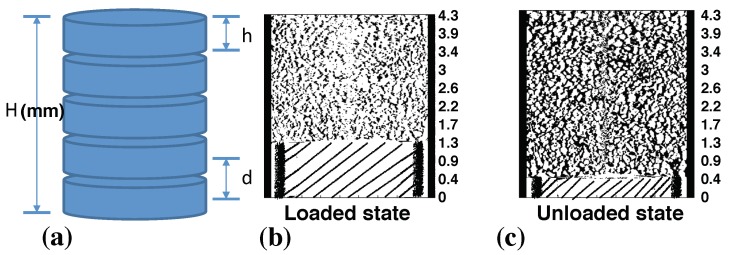
Concepts of porosity calculation: (**a**) in this study, H (height of bed) is 4.23 mm at an unloaded state while h (section height) and d (section distance) are 0.47 mm; (**b**) an example of loaded state; (**c**) an example of unloaded state. Note that area with lines in Figure 3b,c represents the moving punch and the gap between the moving punch and sample holder is 0.05 mm.

Porosity distribution of the investigated volume was calculated for the whole volume and for discretized sections of the volume. In this study, the number of discretized sections varies with the applied load, see Figure 3a. For example, snow volume at unloaded state was divided into 9 sections. In addition, section height and distance were maintained constant during the discretization at all load states. Equation (2) was then used to calculate porosity within each of these sections and for the whole volume.

The density of the snow sample is calculated from the volume of the ice crystals (V${}_{i}$) and the total volume of the snow sample (V${}_{t}$) [13,26]. It is given in the form:

(2) $$\rho_{\mathrm{snow}}=\frac{V_{i}}{V_{t}}*\rho_{ice}\;\;\left[in\;kg/m^{3}\right],$$

where $\rho_{ice}$ = 917 $kg/m^{3}$.

Specific surface area (SSA) is calculated from the volume fraction of ice crystals (V${}_{i}$) and area of ice crystals (A${}_{i}$). It is given in the form:

(3) $$\mathrm{SSA}=\frac{A_{i}}{V_{i}}\;\;\left[\mathrm{in}\;mm^{-1}\right].$$

The calculated SSA, density and porosity values from the micro-CT data at four loading states are presented in Section 4.3.

## 4. Results

The experimental observations from the micro-CT analysis are presented in this section. Distribution of snow grains is given in Section 4.1. Section 4.2 presents the changes in snow microstructure with respect to the applied load. Microstructural parameters for the discretized sections of the snow volume are presented in Section 4.3.

### 4.1. Distribution of Snow Grains

**Figure 4:**
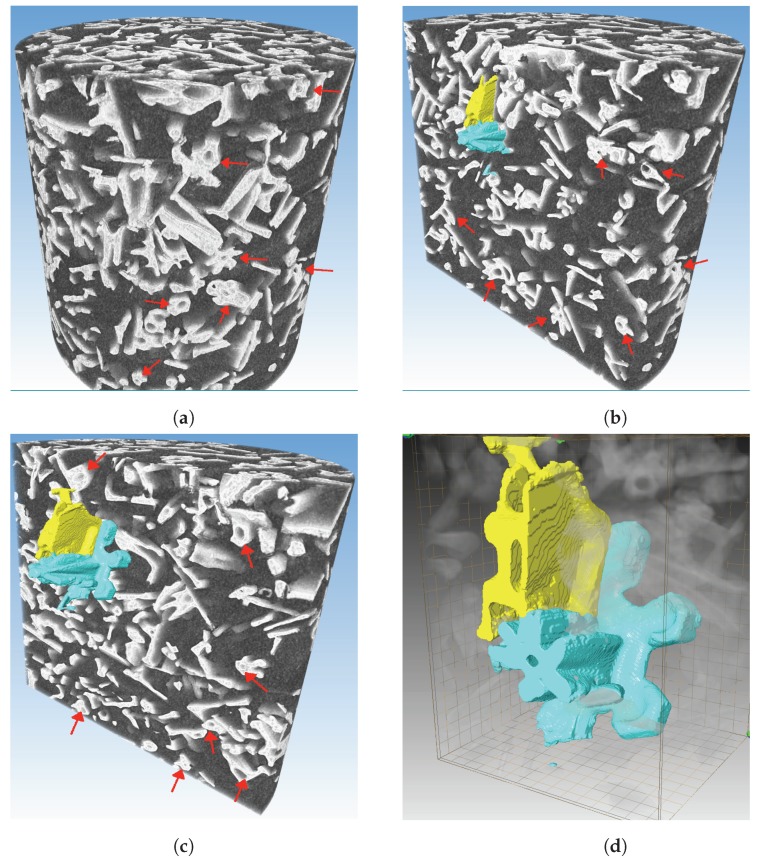
3D images of snow grains distribution: (**a**) scan of the whole investigated snow volume; (**b**) scan of the snow volume across a cross-section and selection of two individual grains; (**c**) slightly deeper cross-section than the one shown in Figure 4b, to focus on the individual grains; (**d**) structure of two individual snow grains. Red arrows in Figure 4 represent an example of the described hollow core structure.

Some examples of the complex 3D morphology of the investigated natural dry snow are presented in Figure 4. The snow sample was composed of ice crystals with significant variations in shape and size. However, moving through the cross sections, the most dominated shapes are needles, capped columns and dendrites, see Figure 4a–c.

There was no possibility for snow grains to grow into different shapes during the scanning time as the snow temperature was kept constant at −15 °C. In addition, there was no presence of water in the pore space between ice crystals. These natural ice crystals observed to be completely non-isotropic in shape and size. A number of the ice crystals observed to have a deep hollow core (see-through tunnel) with variable dimensions (Figure 4d). To study the nature of this hollow core further, two individual ice crystals of different shapes are selected. The structure of these two crystals can be seen at two successive cross-sections in Figure 4b,c. The presented crystals (Figure 4) appeared to have hollow core with closed tip at one end (Figure 4d, the tip was cut). The width of the presented ice crystals was approximately 0.85 mm, and the diameter of the tunnel ranges from 0.06 mm to 0.22 mm Moreover, pore volume of blue and yellow colored crystals (from Figure 4d) was approximately 0.092 $m^{3}$m and 0.051 $m^{3}$m, respectively. One can observe in Figure 4 that a significant number of snow grains have the described hollow core structure, and some examples are shown by red arrows in Figure 4a–c.

### 4.2. Displacement of Snow Grains

**Figure 5:**
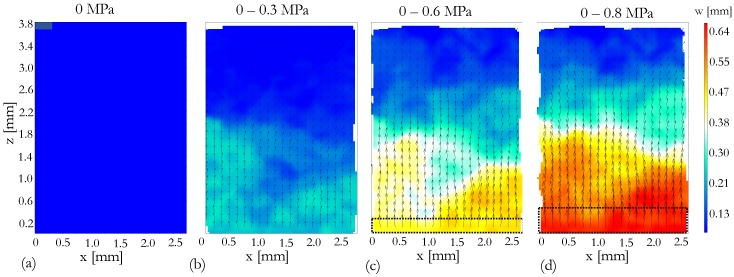
The DVC results for the snow sample at three loaded states, 0.3 MPa, 0.6 MPa and 0.8 MPa. w refers to the displacement in z-direction within the investigated volume. Figure 5a represents the displacement field at unloaded state. Figure 5b,c,d represent the displacement fields between unloaded state and loaded states 0.3 Mpa, 0.6 Mpa and 0.8 Mpa, respectively. Arrows indicate the direction of the displacement field and dashed black lines in Figure 5c,d represent the moving punch.

**Figure 6:**
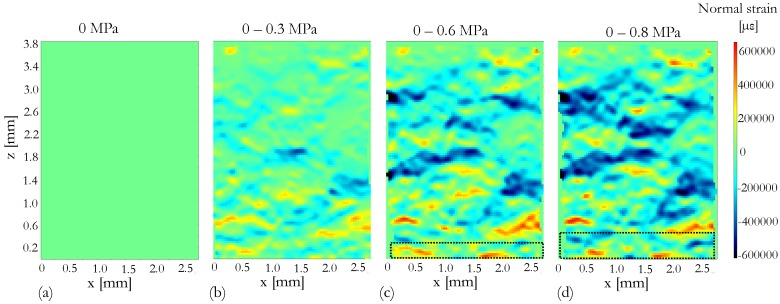
Normal strain fields with respect to the applied stress and dashed black lines in Figure 6c,d represent the moving punch. Figure 6a represents the strain field at unloaded state. Figure 6b,c,d represents the strain fields between unloaded state and loaded states 0.3 Mpa, 0.6 Mpa and 0.8 Mpa, respectively.

Figure 5 and Figure 6 show the displacement and strain fields between unloaded state (0 MPa) and the loaded states at 0.3 MPa (Figure 5b and Figure 6b), 0.6 MPa (Figure 5c and Figure 6c) and 0.8 MPa (Figure 5d and Figure 6d). The spatial resolution of the DVC analysis is limited to the size of the correlation window (sub volume), which is 32 × 32 × 32 voxels. Hence, the DVC analysis is not carried out at granular level, for individual grain tracking.

One can observe in Figure 5 that the measured displacement field at all the loaded states exhibit similar feature of upward compression (in z-direction). This is expected due to the position of the moving punch. In addition, overall displacement of snow volume appeared to be increasing as the applied stress increases, especially close to the moving punch. However, larger amount of deformation can be observed at the center of the sample (in radial direction) compared to the boundaries. This phenomenon may be due to the smaller punch diameter than the sample holder.

Snow grains close to the fixed punch appeared to be insensitive to the applied stress at least up to 0.8 MPa, as no significant displacement is observed, see Figure 5. This may be due to the boundary condition imposed by the fixed punch. At 0.8 MPa of applied stress, significant deformations may be due to breakage of bonds between ice crystals and re-distribution of ice crystals. However, strain fields in Figure 6 shows that the deformations were fairly distributed through out the whole volume where parts of the snow volume experience positive strain field (tensile) while other parts experience negative stain field (compression). One can observe that there is also a part of the snow volume which experiences almost zero deformation. However, tracking of individual grains due to compression needs to be studied further.

**Figure 7:**
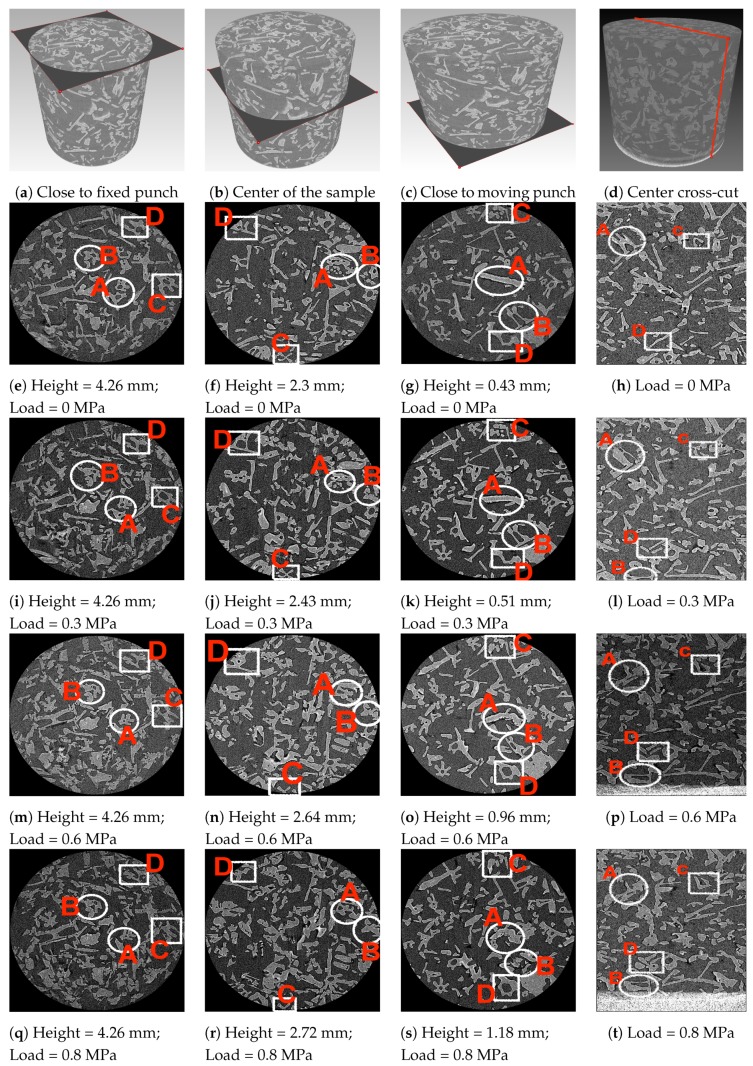
Distribution of ice crystals at four slices of the investigated snow volume at a given load. Note that the compaction is applied from the bottom of the snow volume via moving punch and it is visible in Figure 7p,t.

Figure 7 shows the reconstructed grayscale images of the investigated snow volume at four loading states. Four cross-cuts per loading state are presented so that, behavior of ice crystals can be observed close to the fixed punch (top layer, XY-plane, Figure 7a), in the center of the snow volume (XY-plane, Figure 7b), close to the moving punch (bottom layer, XY-plane, Figure 7c), and center cross-section in z-direction (XZ-plane, Figure 7d). The internal features of the snow volume in Figure 7 are very extensive, therefore four crystals from each image are selected to describe general observations. Note that, the selected cross-cuts for a respective loading were correspond to the same cross-cut (slice), to avoid the rotational effects and to ensure that the observed solid movements of grains are solely due to compression tests.

Figure 7e,i,m,q represent a cross-cut close to the fixed punch (top layers, XY-plane) at load states 0 MPa, 0.3 MPa, 0.6 MPa and 0.8 MPa, respectively. The selected crystals in these four images are named as A, B, C and D. Crystals A and B show that not all the crystals in this cross-cut experience displacement. The dendrite crystals in this case were almost unchanged in terms of shape and size. Crystals C and D experience grains breakage. Analysis of displacement field in Figure 5 show that this part of the snow volume experienced very small displacement. However, observation of individual ice crystals shows a small degree of grain displacement close to the fixed punch, which can be also seen from the strain fields in Figure 6.

Figure 7f,j,n,r represent a cross-cut (XY-plane) at the center of the snow volume at all load states. The selected crystals in these four images are named as A, B, C and D. Crystals A and B tend to move closer to each other and form well connected grains. Crystals C and D, experience re-distribution due to the formation of new crystals, and one can observe that crystals C and D moves out of the scan volume as the compaction increases.

Figure 7g,k,o,s represent a cross-cut (bottom layers, XY-plane) close to the moving punch at all load states. The selected crystals in these four images are named as A, B, C and D. Crystals A and B tend to experience breakage of grains bond due to compaction, while crystals C and D tend to form into new crystals. As this part of the snow volume experienced the compaction directly from the punch, crystals observed to be deformed significantly via bond breakage. This was already observed from the displacement field in Figure 5.

Figure 7h,l,p,t represent a center cross-cut (XZ-plane) in z-direction at all load states. The selected crystals in these four images are named as A, B, C and D. Crystals A, C and D tend to experience recrystalization as the applied stress increases. Moreover, one can observe the moving punch in Figure 7p,t. As the punch compact the snow surface, crystals tend to appear within the VOI (for example, crystal B).

### 4.3. Porosity Measurements

**Figure 8:**
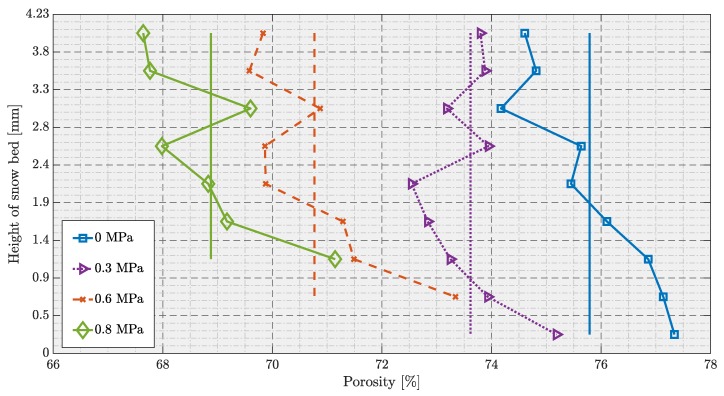
Porosity distributions for the discretized sections of the investigated snow volume. The calculated density values of the snow volume at each load state are given (in units $kg/m^{3}$).

Figure 8 shows the calculated porosity distribution for the discretized sections of the snow volume and the whole volume. The straight lines in Figure 8 represent the average porosity values for the whole volume at a given load state. Note that the moving punch applied compaction from the bottom, as shown in Figure 3b,c. The calculated density and SSA are given in Table 1.

One can observe that the porosity of the whole snow volume linearly decreases as the applied compression increases. Under compaction, the ice crystals tend to move closer resulting in densification where the ice crystals re-distribute into the pore space. Note that the moving punch is excluded from the data shown in Figure 8, which can be observed from the reduction of data points as the investigated snow volume experienced compaction.

There is a distinct vertical heterogeneity can be observed for the porosity distribution in the axial direction, see Figure 8. At an unloaded state, the initial snow sample exhibits a higher porosity (77.5%) at the bottom compared to the top (74.5%) of the sample. This may be due to the preparation of the snow sample. The sample holder was pushed straight into the dry natural snow and shaved off the excess snow from the top with a sharp spatula, producing a flat and smooth surface. The described sample preparation appeared to force the grains to move closer at the top of the sample holder, which resulted in around 3% of porosity variation between top and bottom layers.

At 0.3 MPa loading stage, this vertical heterogeneity in porosity distribution between the top (74%) and bottom (75%) layers was reduced to around 1%. This may be due to the initial compression, which forces the grains to re-arrange. Furthermore, there was more than 6 hours of settling process during the scan at unloaded state. It may also be possible that the bottom layers experience the weight of top layers due to settlement thus forcing the distribution of ice crystals into the pore space.

At 0.6 MPa and 0.8 MPa load stages, the vertical heterogeneity in porosity distribution between the top (73.5% & 71%, respectively) and bottom (70% & 67.5%, respectively) layers was then re-increased to around 3%. As described in Figure 7, snow grains at higher loads experience bond breakage and grain re-arrangement. Table 1 further shows that the overall density increases with compression while the SSA decreases.

## 5. Discussions

General observations can be deduced from the results of this initial study. The present study shows that displacement of snow’s internal structures during compaction can be investigated from the micro-CT data coupled with the DVC and porosity analysis. Embodied in this study are the vital observations that (1) the majority of snow grains have deep hollow core, (2) displacement of snow grains due to grains breakage and recrystallization, and (3) vertical heterogeneity in porosity distribution of the snow sample in response to compaction.

The growth of ice crystals depends on the temperature and humidity [27]. Shimada and Ohtake [28], Wergin et al. [29] and Riche et al. [30] reported that ice crystals grow as columns and needles around −5 °C and as plates and dendrites around −15 °C. This may explain the complex structure of the investigated dry natural snow as colder temperatures higher up in the atmosphere produces dendritic snow and needles are formed during the precipitation close to ground.

The complex microstructure of the investigated snow volume shows the inter-granular structural changes due to compaction. Compaction of snow by applying micro-mechanical uniaxial load forces the individual ice crystals to move closer via grain sintering and bond breakage. This process is known as pressure sintering [31], which results in rounded and less complex microstructure of snow. Wilkinson [32] detailed the influence of pressure sintering on snow microstructure and its mechanical properties. The sintering process in a snow pack was observed previously [33,34], where snow coarsening was forced due to loading and liquid water content. A common observation of the snow compaction between the tomography data and our previous works, is that the majority of displacement of snow grains occur at the near-surface layers. Gubler [35], Szabo and Schneebeli [36] reported that they observed similar behavior of snow microstructure during compaction with respect to temperature and time. Moreover, Schleef et al. [16] observed the sintering effect during the relaxation phase of a snow sample, which has similar microstructural characteristics as the one presented in this study. Major observations from their work are based on variations in strain rates and microstructural properties during the relaxation stage. They also discussed about a critical point during higher compression rates where a snow structure can be collapsed and rearranges into more compact structure.

The tomography data presented in this initial study was useful in terms of higher resolution measurements, observation of hollow core structure and analysis of porosity distribution. However the study is also limited by the long measurement time, lack of stress relaxation measurements, correlation window size for DVC analysis and the sample size. In principle, this initial study can be further extended in future to analyze different snow types under the influence of loading and isothermal metamorphism conditions. In addition, the selected VOI (refer to Section 2.2) must consider the whole volume to observe the edge effects. Figure 7 shows that some of ice crystals move out of the scan volume during compaction due to the finite size of the VOI, where the relevant information about the re-distribution of these ice crystals is missing.

Furthermore, the sample preparation appears to play a major role especially when it comes to tomography measurements. Ebner et al. [12], Zermatten et al. [37] performed structural analysis of snow samples by exposing to an advective airflow, and further observed porosity distribution, coarsening rate and settling of snow. In addition, Ebner et al. [12] reported that there is no correlation between the porosity distribution and settling of snow. However, a clear correlation is observed in this study where, porosity distribution at 0.3 MPa load stage appeared to be due to the snow settlement, see Figure 8.

The elastic characteristics of the described hollow core feature can be further investigated in future experiments. The observations from this study to understand the microstructural changes in snow grains and properties in response to compression tests, may be helpful to investigate the snow mechanics. Further experiments considering the limitations, can be advantageous in various fields of snow research. In specific, the observations in this study can be useful to understand snow mechanics for winter tire testing, ski track preparation and avalanche prediction. When snow is compressed, friction between the snow and tire decreases, which can be investigated further based on the presented study. For the ski tracks and avalanche, the investigation in this study helps to understand the snow quality and snow pack stability.

## 6. Conclusions

An X-ray micro-tomographic (micro-CT) measurements coupled with digital volume correlation and microstructural study are performed to investigate the microstructure of dry natural snow. The three-dimensional (3D) reconstruction of snow microstructure is essential to understand its metamorphism, and physical and mechanical properties of a snowpack. This initial study allows for an observation of the changes in internal microstructure during compression tests. Moreover, displacement and strain fields of the snow volume and microstructural properties such as porosity, density and specific surface area (SSA) are determined. The porosity distribution of the whole snow volume decreases with an increase in level of compaction. In addition, the analysis of vertical heterogeneity in porosity distribution shows the characteristics of snow sample preparation and settling of snow. The observations in this study further showed that the majority of ice crystals in the investigated snow volume has a deep hollow core, irrespective of the shape and size of the crystal. The DVC analysis showed the displacement field in response to compaction, while the two-dimensional (2D) cross-cuts of the snow volume showed the grain re-arrangement and bond breakage due to compaction. The presented techniques may be useful to determine stress-strain response of snow for better understanding of the various snow layer transitions.

## Figures and Tables

**Table 1:** Microstructural parameters under loading conditions.

Stress (MPa)	Density (kg m${}^{-3}$)	SSA (mm${}^{-1}$)
0	224	66
0.3	243	64
0.6	265	62
0.8	284	60
